# Dynamic prediction of long-term survival in patients with primary gastric diffuse large B-cell lymphoma: a SEER population-based study

**DOI:** 10.1186/s12885-019-5993-6

**Published:** 2019-09-03

**Authors:** Ju-Li Lin, Jian-Xian Lin, Ping Li, Jian-Wei Xie, Jia-bin Wang, Jun Lu, Qi-Yue Chen, Long-long Cao, Chang-Ming Huang, Chao-Hui Zheng

**Affiliations:** 1 Department of Gastric Surgery, Fujian Medical University Union Hospital, No.29 Xinquan Road, Fuzhou, 350001 Fujian Province China; 2 Department of General Surgery, Fujian Medical University Union Hospital, Fuzhou, Fujian Province China; 3 Key Laboratory of Ministry of Education of Gastrointestinal Cancer, Fujian Medical University, Fuzhou, Fujian Province China

**Keywords:** Primary gastric diffuse large B-cell lymphoma, Cancer-specific survival rate, Nomogram, Web survival rate calculator, Dynamically predict

## Abstract

**Background:**

This study investigated a large number of patients to develop a predictive nomogram for survival and a web-based survival rate calculator that can dynamically predict the long-term survival of patients with primary gastric diffuse large B-cell lymphoma.

**Methods:**

A total of 2647 patients diagnosed with primary gastric diffuse large B-cell lymphoma from 1998 to 2014 were extracted from the SEER database. We used the Lasso Cox regression model to identify independent risk factors for long-term survival and to develop a predictive nomogram for survival and a web-based survival rate calculator.

**Results:**

The median (mean) follow-up time was 30 months (52.8 months). Cancer-specific survival rates decreased with time, while the 5-year conditional survival increased with time. Cancer-specific deaths were not constant. Cancer-specific deaths of patients within the first 2 years were high, while the risk remained relatively constant after 2 years. The independent risk factors included surgery, chemotherapy, tumor stage and age, according to the Lasso Cox regression analysis. We developed a predictive nomogram and a web-based survival rate calculator (https://linjuli1991.shinyapps.io/dynnomapp/). The calibration plot suggested that the actual value exhibited good agreement with the predicted value.

**Conclusions:**

We found that patients with primary gastric diffuse large B-cell lymphoma had a high risk of death during the first 2 years. Additional active follow-up strategies should be provided during this period. This is the first study to develop a predictive nomogram and a web-based survival rate calculator that can provide evidence for individual treatment and follow-up.

**Electronic supplementary material:**

The online version of this article (10.1186/s12885-019-5993-6) contains supplementary material, which is available to authorized users.

## Background

The National Comprehensive Cancer Network (NCCN) Guidelines use Ann Arbor staging of primary diffuse large B-cell lymphoma to guide clinical treatment and follow-up [1]. As a general staging system for non-Hodgkin’s lymphoma (NHL), the Ann Arbor staging system considers the location of lymph node dissemination as the basis for staging [2]. Other factors that may affect long-term survival, such as age and depth of tumor invasion, are not included. The Ann Arbor staging system is not considered the best staging system for primary gastric diffuse large B-cell lymphoma [3]. Therefore, this study investigated a large number of patients to develop a predictive nomogram for survival and a web-based survival rate calculator that can dynamically predict the long-term survival of primary gastric diffuse large B-cell lymphoma patients. Furthermore, we also investigated the probability of survival increasing over time based on survival times previously accumulated to guide treatment and follow-up strategies.

## Methods

### Patient selection

The inclusion criteria which has been reported in our previous study [4] were as follows: A case listing session was created from the Surveillance, Epidemiology, and End Results (SEER) program using SEER*Stat 8.2.1 (http://seer.cancer.gov/seerstat). A total of 2647 patients diagnosed with primary gastric diffuse large B-cell lymphoma from 1998 to 2014 were extracted from the SEER database. Patients diagnosed between January 1998 and December 2014; Ann Arbor [8] staging codes ([EOD] 10 - extent [1988–2003]; Collaborative Stage [CS] extension [2004+] stage; I and II, and stage III and IV ([ICD-O-3 topography); [pathological diagnosis of code], 16.0–16.9) ([ICD-O-3] 9680/3); the operation code (RX Summ--Surg Prim Site (1998+), 30–80); chemotherapy recode: chemotherapy code (yes, no/unknown)); and radiotherapy (radiation recode) code. The exclusion criteria were as follows: Ann Arbor staging was unknown, patients younger than 18 years old, multiple tumors (first malignant primary indicator), deaths that were not tumor-related (SEER “other cause of death” classification), patients who die from complications of chemotherapy or radiotherapy and patients who died within 30 days.

### Statistical analysis

Statistical analyses were performed using SPSS software (version 22.0) for Windows (SPSS Inc., Chicago, IL), R software (version 3.4.0) (http://www.r-project.org/) and Rstudio software 1.1.383 (https://www.rstudio.com/). Cumulative survival rates were estimated using the Kaplan-Meier method and compared using the log-rank test. The cancer-specific survival hazard curve was plotted using the life table. We used the Lasso Cox regression model to identify independent risk factors for long-term survival [5]. The “glmnet” package was used to perform the Lasso Cox regression model analysis. Nomogram and calibration plots were generated using the “rms package” of R software. Discrimination was evaluated using a concordance index (C-index). A calibration plot was generated to explore the performance characteristics of the nomogram. In addition, we applied a bootstrapped resample with 1000 iterations to verify the accuracy of the nomogram. The “shiny” and “DynNom” packages were used to generate a web-based survival rate calculator, which can dynamically predict cancer-specific survival rates (https://www.shinyapps.io/). Conditional survival [6–8] estimates were calculated as the probability of survival for an additional 5 years (CS_5_), given that the patient had survived for 1, 2, 3, 4, or 5 years, and were calculated using the following formula: CS_5_ = S_(X + 5)_/S_(X)_: For example, 5-year CS among patients who had survived 3 years from the date of surgery was calculated by dividing the 8-year survival rate by the 3-year survival rate. Two-sided *P*-values less than 0.05 were considered significant.

## Results

### Baseline characteristics of patients

The baseline clinical characteristics of the patients are shown in Table 1. Among the patients, 1240 (46.8%) were < 65 years, 608 (23%) were 65–74 years old, and 799 (30.2%) were > 75 years. The patients included 1539 (58.1%) men and 1108 (41.9%) women. A total of 2038 patients (77%) were white, and the other 609 patients were non-white (23%). A total of 646 (24.4%) patients had tumors located in the upper part of the stomach, 666 (25.2%) patients had tumors in the lower part of the stomach, 292 patients (11%) had tumors in the whole stomach, and for 1043 patients (39.4%) the location was unknown. The Ann Arbor stages were distributed as follows: 1167 (44.1%) cases were stage I, 582 (22%) cases were stage II, 211 (8%) cases were stage III, and 687 (26%) cases were stage IV. A total of 502 (19%) patients were treated with radiotherapy, and 2145 (81%) patients did not receive radiotherapy. A total of 2075 (78.4%) patients were treated with chemotherapy, and 572 (21.6%) patients did not receive chemotherapy or chemotherapy status was unknown. A total of 275 (10.4%) patients were treated with surgery, and 2372 (89.6%) patients did not undergo surgery.

**Table 1:** Baseline characteristics of the patients

Variable	2647 (%)
Age	
< 65	1240 (46.8%)
65–74	608 (23.0%)
> 74	799 (30.2%)
Gender	
Male	1539 (58.1%)
Female	1108 (41.9%)
Race	
White	2038 (77.0%)
Other	609 (23.0%)
Tumor location	
Upper third	646(24.4%)
Mid and low third	666(25.2%)
Overlapping stomach	292 (11.0%)
Stomach (NOS)	1043(39.4%)
Ann Arbor stage	
I	1167 (44.1%)
II	582 (22.0%)
III	211 (8.0%)
IV	687 (26.0%)
Radiation	
Yes	502 (19.0%)
No	2145 (81.0%)
Chemotherapy	
Yes	2075 (78.4%)
No/Unknown	572 (21.6%)
Surgery	
Yes	275 (10.4%)
No	2372 (89.6%)

### Long-term patient survival

**Fig. 1:**
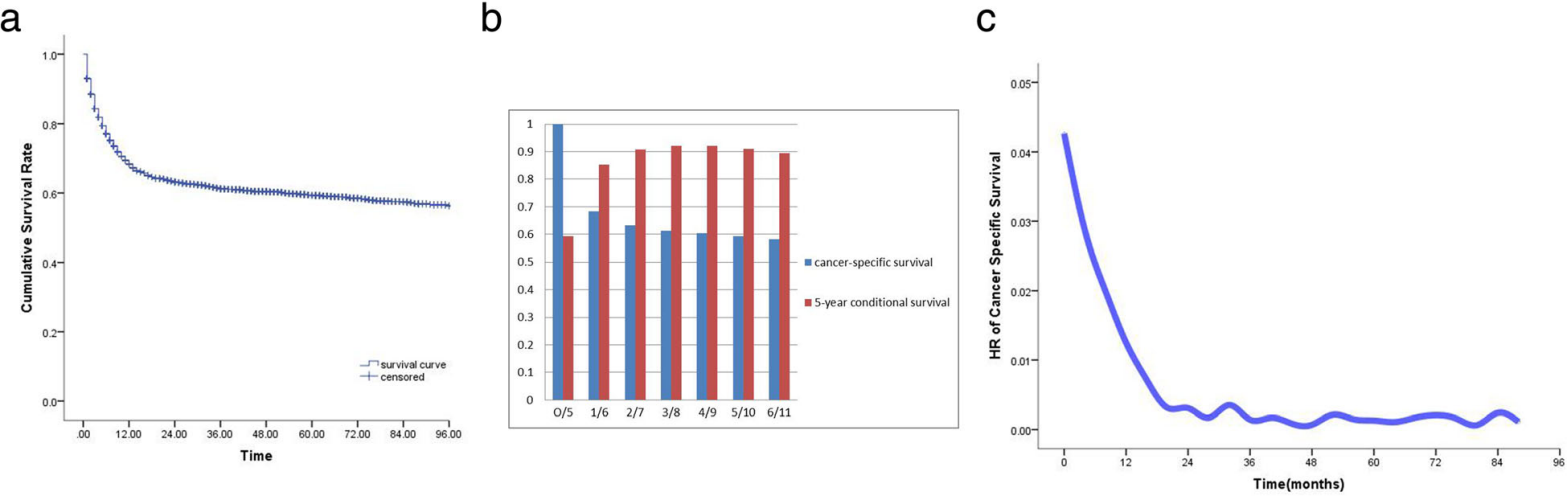
**a** Kaplan-Meier curve for cancer-specific survival of the patients. **b** 5-year conditional survival (CS_5_). **c** Cancer-specific survival hazard curve

The median (mean) follow-up time was 30 months (52.8 months) for all the patients, and the cancer-specific survival rate is shown in Fig. 1a. The cancer-specific survival rates for 1, 2, 3, 4 and 5 years were 68.4, 63.2, 61.2, 60.4 and 59.3%, respectively, indicating a decreasing trend over time. The CS rates for 1, 2, 3, 4 and 5 years were 85.2, 90.8, 92.1 and 92% and 91.1%, respectively. The CS_5_ rate increased over time (Fig. 1b). The cancer-specific survival risk hazard is shown in Fig. 1c, which indicates that cancer-specific death of primary gastric diffuse large B cells is not constant. The rate of cancer-specific death within the first 2 years was high, while the risk remained relatively constant after 2 years. 5-year cancer specific survival rate of patients is 59.3% and 5-year overall survival rate of patients 52.4% (Additional file 1: Figure S1). 5-year cancer specific survival rate of patients who receive chemotherapy is 65.1% while no chemotherapy is 38.1%% (Additional file 2: Figure S2). 5-year cancer specific survival rate of patients who receive partial gastrectomy is 63.3% total gastrectomy is 64.9% (Additional file 3: Figure S3).

### Analysis of long-term patient survival

**Fig. 2:**
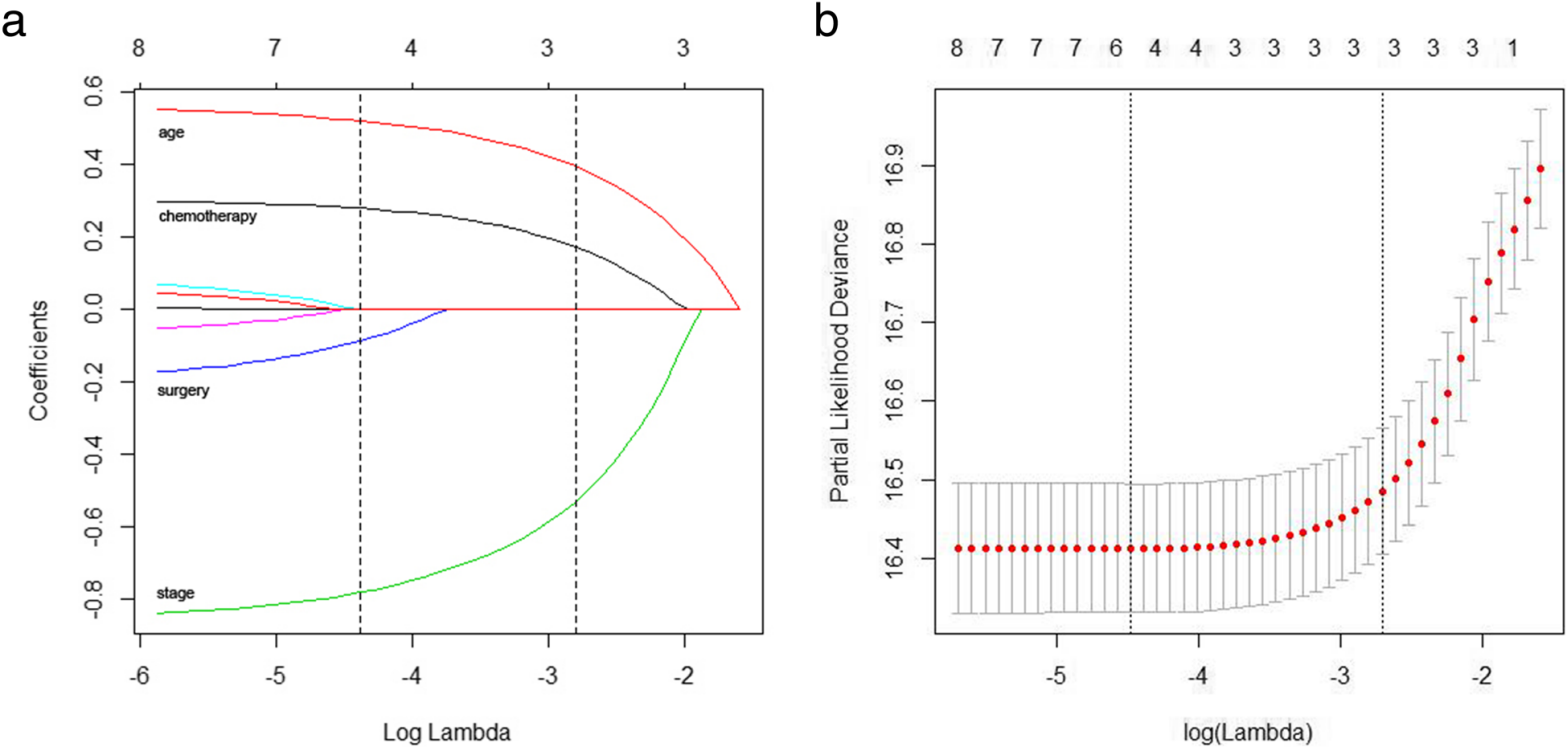
**a** Lasso regression coefficients. **b** Lasso cross-validation.

The independent risk factors affecting the long-term survival of patients were determined by Lasso Cox regression analysis. Each colored line represents a variable in the model. With increases in λ, the coefficient of each variable decreased. When the λ was optimal, the coefficients of some variables were compressed to 0; therefore, the variables that were not 0 were retained, and variable selection was performed. The blue, green, black and red solid lines that cross the dashed line in Fig. 2a indicate the independent prognostic factors, including surgery, chemotherapy, tumor stage and age, respectively. The minimum cross-validation error was 0.0114, and the maximum cross-validation error was 0.067 (Fig. 2b), which is within the range of standard error.

### Predictive nomogram for cancer-specific survival

**Fig. 3:**
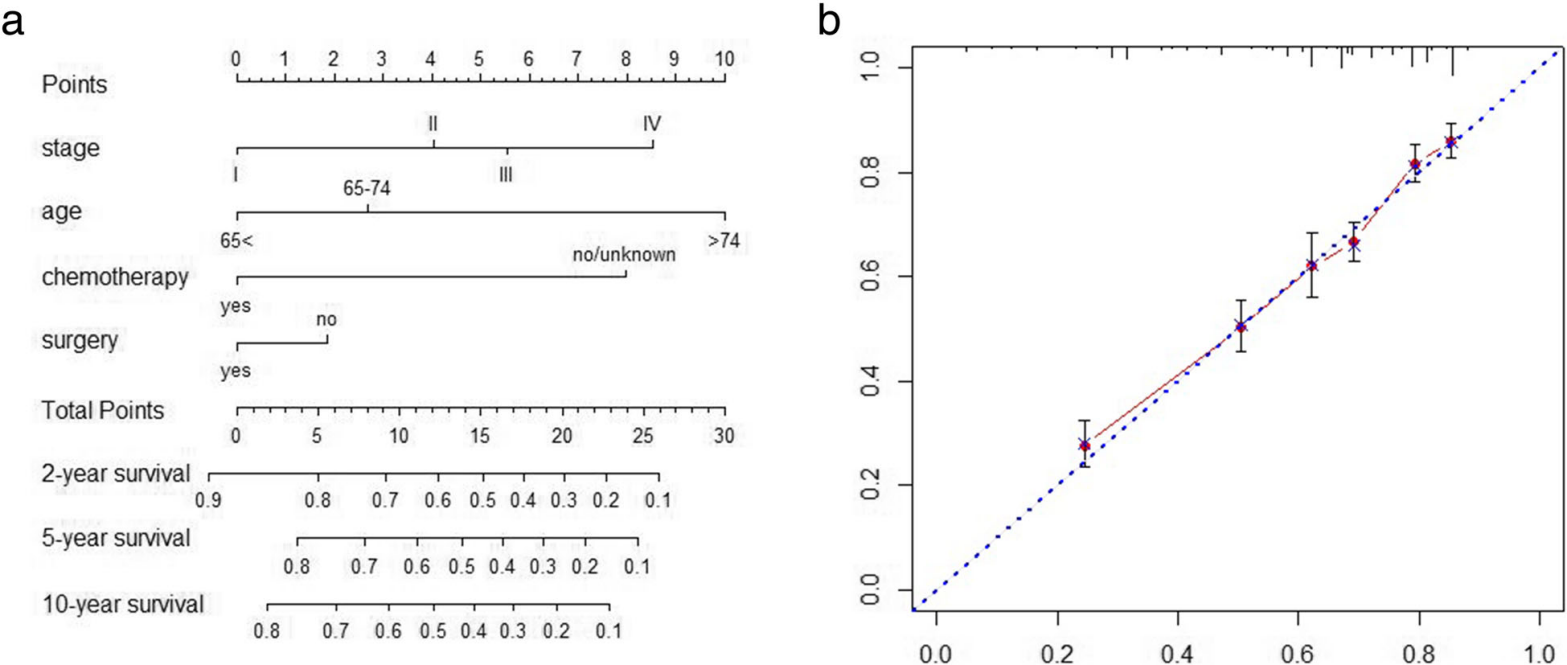
**a** Predictive nomogram for cancer-specific survival (C-index 0.714). **b** Calibration plot of 5-year cancer-specific survival

Figure 1 illustrates the predictive nomogram established for cancer-specific survival rates based on independent risk factors such as Ann Arbor stage, age, surgery and chemotherapy. The discriminative ability of the nomogram was superior to that of the Ann Arbor stages (C-index of 0.714 vs. 0.56, *P* < 0.01). The calibration plot (Fig. 3b) suggested that the actual value exhibited good agreement with the predicted value.

### Web-based survival rate calculator

**Fig. 4:**
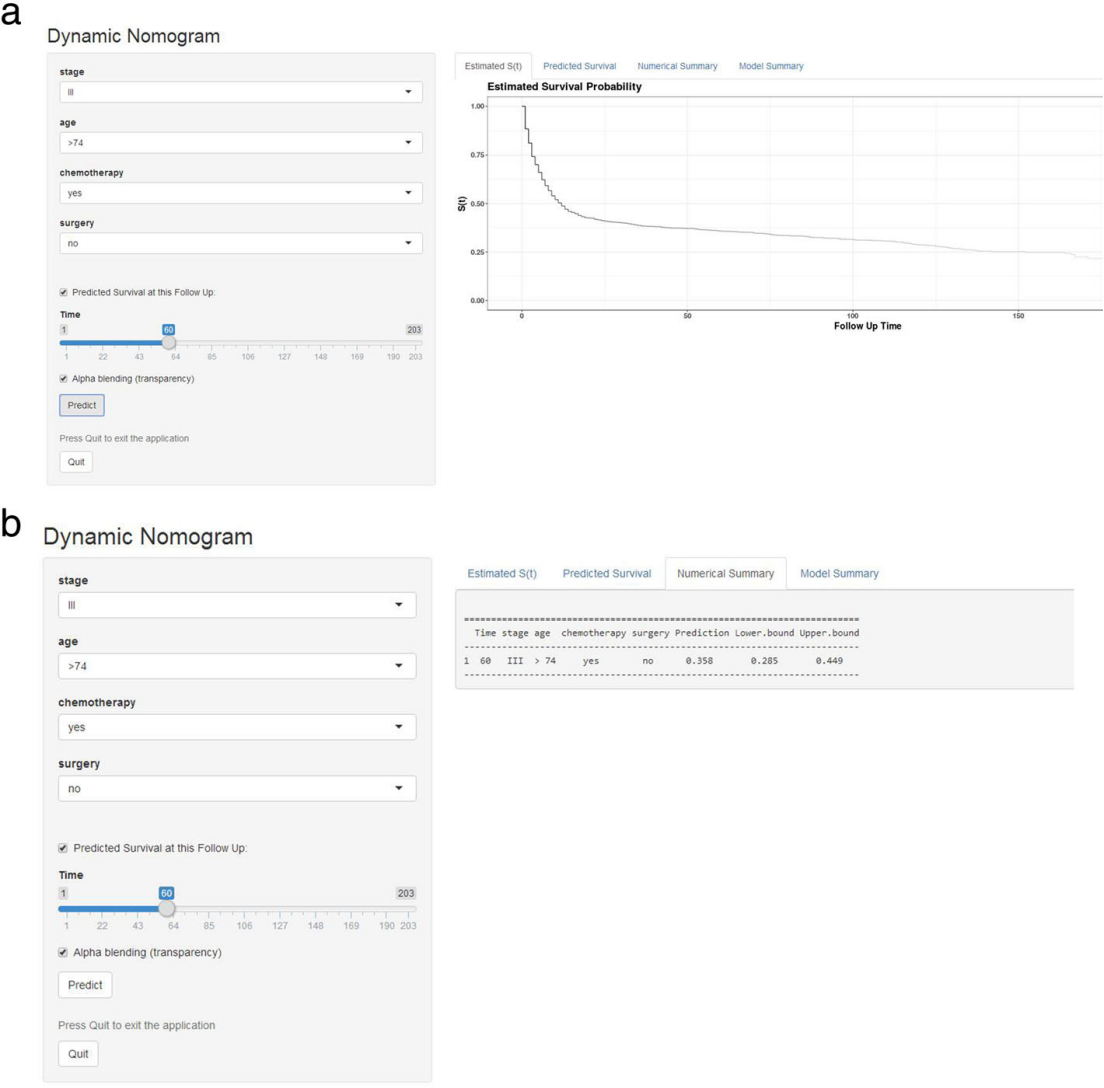
**a** Patient with an Ann Arbor stage of III, age > 74 years, no surgery, who received chemotherapy according to the web survival rate calculator (95% CI 28.5–44.9%). **b** 95% confidence interval according to the web survival rate calculator

According to the above results, we established a dynamic web-based survival rate calculator (https://linjuli1991.shinyapps.io/dynnomapp/) to predict the long-term survival of patients with primary diffuse large B cells based on a nomogram (Fig. 4a). The calculator can individually predict the survival of patients according to their clinical characteristics. For example, the 5-year cancer-specific survival rate is approximately 35.8% (95% CI 28.5–44.9%) for patients with Ann Arbor stage of III, aged > 74 years, without surgery and chemotherapy (Fig. 4b).

## Discussion

Precision medicine rapidly developed in recent years. Clinicians must generate individualized treatment and follow-up strategies for patients, which requires more precise and convenient survival models. Nomograms integrate tumor stage and multiple prognostic factors into a simple and practical tool that has been widely used to predict the long-term survival of patients with malignant tumors. The accuracy is usually superior to traditional tumor staging systems [9–13]. In addition, to increase the convenience of predictive models, some scholars have used a web-based survival rate calculator [13–15] to predict the long-term survival of cancer patients. Primary gastric diffuse large B-cell lymphoma is a rare malignant tumor with an incidence of less than 5% of gastric malignant tumors [16]. A nomogram to predict survival and a web-based survival rate calculator for primary gastric diffuse large B-cell lymphoma have not been previously reported. Therefore, this study examined 2647 patients in the SEER database to analyze factors that affect long-term survival and used a conditional nomogram web-based survival rate calculator to dynamically predict the prognosis of primary gastric diffuse large B-cell lymphoma with different risk factors to determine individual treatment and follow-up strategies.

The traditional cancer-specific survival rate of tumors is an important basis for guiding treatment, follow-up and surveillance. However, the risk of cancer-specific-death is not constant and often changes over time. The risk of recurrence and death is usually the highest during the first few years after treatment, while the survival rate tends to be constant over time. Therefore, traditional survival rates exhibit a significant deficiency for dynamically evaluating the survival of tumor patients. CS [6–8, 17–19], which accounts for survival time already accumulated, provides a more “dynamic” estimate of the risk of death over time. It is an important tool for guiding long-term follow-up. In addition, the NCCN guidelines recommend that patients with diffuse large B-cell lymphoma should be followed up every 3–6 months for 5 years. This study combined cancer-specific survival rates, CS_5_ and hazard curves; the risk of death was high during the first 2 years after treatment and remained relatively constant after 2 years, which suggested that primary gastric diffuse large B-cell lymphoma patients should receive additional active follow-up during the first 2 years after treatment.

In addition, Lasso Cox regression analysis was used to analyze the prognostic risk factors. In contrast to the traditional stepwise Cox regression analysis, Lasso Cox regression can analyze all independent variables at the same time rather than using gradual processing. It is a new method for variable selection and shrinkage in a Cox proportional hazards model. It minimizes the log partial likelihood subject to the sum of the absolute values of the parameters being bound by a constant. Due to the nature of this constraint, it minimizes coefficients and produces some coefficients that are exactly zero. Therefore, it reduces the estimation variance while providing an interpretable final model. Simulations have indicated that Lasso regression is more accurate than stepwise selection [5, 13]. According to the Lasso Cox regression analysis, we found that age, tumor stage, chemotherapy and surgery are independent risk factors for long-term survival. Due to the overall poor functionality, low resilience and short life expectancy, the survival rate of elderly patients was relatively low. Previous studies [20, 21] have suggested that tumor stage is an important risk factor for long-term survival, and the survival rate of early-stage patients is higher than that of patients with advanced stage tumors. Chemotherapy is one of the main treatment methods according to the guidelines for lymphoma published by the Japanese Gastric Cancer Association (JGCA) and the NCCN Guideline [1, 22]. This treatment plays an important role in the prognosis of patients. Surgical indications are limited to bleeding, acute perforation, pyloric obstruction, or contraindications to chemotherapy during the course of non-surgical treatment. Studies [23, 24] have shown that combined chemotherapy and surgery could not significantly improve the long-term survival of patients. However, some studies [21, 25] have found that patients who received surgery combined with chemotherapy had significantly better prognoses than those who received chemotherapy alone. In this study, Lasso Cox regression analysis showed that surgical treatment was an independent factor for survival.

In this study, we try to incorporate factors which may have an impact on survival. Many studies [9, 10, 12, 26, 27] have suggested that nomograms may be useful tools for predicting long-term prognoses. Using a simple graphical representation, nomograms enable the incorporation of multiple relevant clinical predictors and can be applied to an individual patient’s combination of relevant clinical factors. Our predictive nomogram included age, Ann Arbor stage, chemotherapy and surgery and was superior in assessing prognoses to the Ann Arbor stage alone (C-index 0.714 vs. 0.56, *P* < 0.01). Although this nomogram was highly accurate, it may be difficult to use in clinical settings due to the need to perform manual calculations. Therefore, our team, for the first time, developed a web-based survival rate calculator based on prediction nomograms of primary gastric diffuse large B lymphoma. In this study, the 5-year survival rate of a patient with an Ann Arbor stage of III, aged > 74 years, without surgery who received chemotherapy was 35.8% (95% CI 28.5–44.9%). The prediction accuracy of the web-based calculator was higher than that of the nomogram according to the 95% confidence interval. The web-based calculator allows better visualization than other web-based survival rate calculators. It can dynamically predict the cancer-specific survival rate of patients at different time points and help to identify patients at high risk of cancer-specific death.

In this study, we compared overall survival and cancer specific survival of patients. 5-year CSS (59.3%) was significant higher than 5-year OS (52.4%). In order to explore the impact of the disease on long-term survival of patients and make the purpose of this study clear, we included CSS as the end point of the study. The majority within 30 days is most caused by surgery or complications. Cancer-specific survival can be better studied by excluding patients who died within 30 days.

Our study had several limitations which has been reported in our previous study [4] were as follows: A data selection bias existed because this study was analyzed in a retrospective manner. The generalizability of the results also requires further validation with external data. We try to incorporate factors which may have an impact on survival, but the SEER database was lacking of detail information of adjuvant therapy, detailed pathological types, gene expression and postoperative complications. We are unable to further analyze the impact of rituxan, non-anthracycline chemotherapy, specific dosage of radiation, different pathological types, gene types and complications on the survival of patients. And it can to be confirmed by more prospective multi-center clinical research data. However, primary gastric diffuse large B-cell lymphoma is a relatively rare disease, and studies with large numbers of patients are lacking. This study may serve as a basis for subsequent research. The web-based survival calculator can be updated and further validated externally.

## Conclusion

In conclusion, our study investigated a large number of patients and analyzed clinical characteristics and prognostic factors. We found that patients with primary gastric diffuse large B-cell lymphoma had a high risk of death during the first 2 years. Active follow-up strategies should be increased during this period. This is the first study to develop a predictive nomogram and a web-based survival rate calculator that can provide evidence for individual treatment and follow-up strategies.

## Additional files

Additional file 1: **Figure S1.** 5-year cancer specific survival rate of patients is 59.3% and 5-year overall survival rate of patients 52.4%. (JPG 27 kb)

Additional file 2: **Figure S2.** 5-year cancer specific survival rate of patients who receive chemotherapy is 65.1% while no chemotherapy is 38.1%%. (JPG 27 kb)

Additional file 3: **Figure S3.** 5-year cancer specific survival rate of patients who receive partial gastrectomy is 63.3% total gastrectomy is 64.9%. (JPG 25 kb)

## Data Availability

The data that support the findings of this study are available from the corresponding author upon reasonable request.

## Abbreviations

CS_5_: 5-year conditional survival
CSS: Cancer-specific survival
JGCA: Japanese Gastric Cancer Association
NCCN: National Comprehensive Cancer Network
NHL: Non-Hodgkin’s lymphoma
OS: Overall survival
SEER: Surveillance, Epidemiology, and End Results
