# Supplementary figures and images for: Dynamic prediction of long-term survival in patients with primary gastric diffuse large B-cell lymphoma: a SEER population-based study
Source: BMC Cancer. 2019 Sep 3;19:873. doi: 10.1186/s12885-019-5993-6 (PMC6724291; doi:10.1186/s12885-019-5993-6)

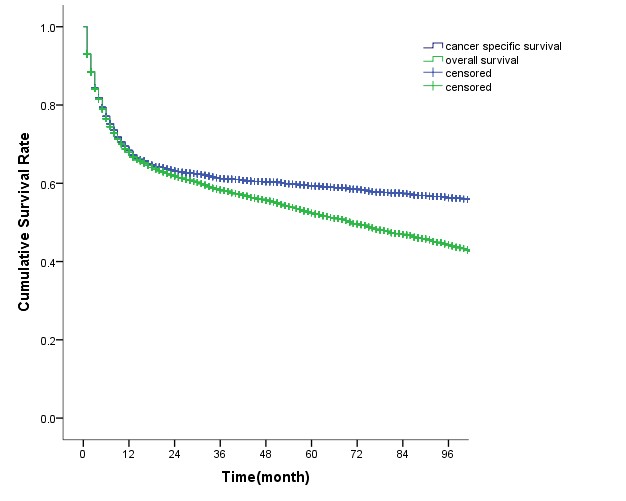

Supplement: Supplementary file 1 — Figure S1. 5-year cancer specific survival rate of patients is 59.3% and 5-year overall survival rate of patients 52.4%. (JPG 27 kb) [file 12885_2019_5993_MOESM1_ESM.jpg]

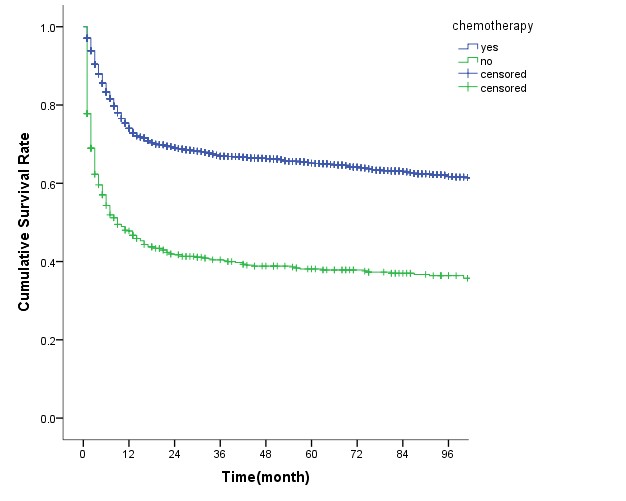

Supplement: Supplementary file 2 — Figure S2. 5-year cancer specific survival rate of patients who receive chemotherapy is 65.1% while no chemotherapy is 38.1%%. (JPG 27 kb) [file 12885_2019_5993_MOESM2_ESM.jpg]

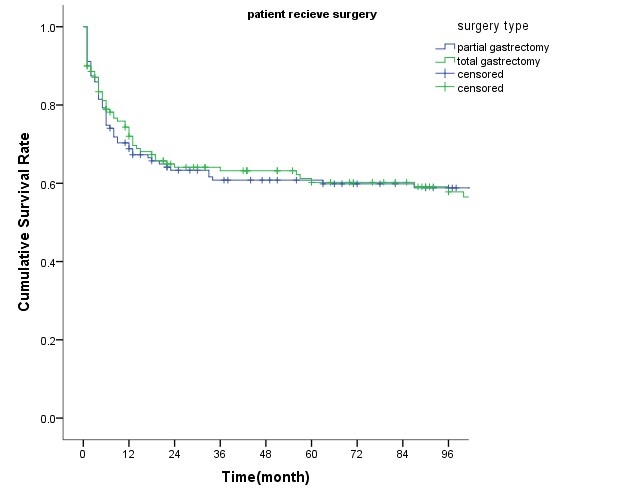

Supplement: Supplementary file 3 — Figure S3. 5-year cancer specific survival rate of patients who receive partial gastrectomy is 63.3% total gastrectomy is 64.9%. (JPG 25 kb) [file 12885_2019_5993_MOESM3_ESM.jpg]
